# Injection Practices and Sexual Behaviors Among Persons with Diagnosed HIV Infection Who Inject Drugs — United States, 2015–2017

**DOI:** 10.15585/mmwr.mm6830a1

**Published:** 2019-08-02

**Authors:** Sharoda Dasgupta, Yunfeng Tie, Ansley Lemons, Kathleen Wu, Janet Burnett, R. Luke Shouse

**Affiliations:** ^1^Division of HIV/AIDS Prevention, National Center for HIV/AIDS, Viral Hepatitis, STD, and TB Prevention, CDC; ^2^Chenega Professional and Technical Services, Chesapeake, Virginia.

During 2016, 6% of persons in the United States who received a diagnosis of human immunodeficiency virus (HIV) infection had their HIV infection attributed to injection drug use (*1*). Injection practices and sexual behaviors among HIV-positive persons who inject drugs, such as injection equipment sharing and condomless sex, can increase HIV transmission risk; nationally representative estimates of the prevalences of these behaviors are lacking. The Medical Monitoring Project (MMP) is an annual, cross-sectional survey that reports nationally representative estimates of clinical and behavioral characteristics among U.S. adults with diagnosed HIV (*2*). CDC used MMP data to assess high-risk injection practices and sexual behaviors among HIV-positive persons who injected drugs during the preceding 12 months and compared their HIV transmission risk behaviors with those of HIV-positive persons who did not inject drugs. During 2015–2017, approximately 10% (weighted percentage estimate) of HIV-positive persons who injected drugs engaged in distributive injection equipment sharing (giving used equipment to another person for use); nonsterile syringe acquisition and unsafe disposal methods were common. Overall, among HIV-positive persons who injected drugs, 80% received no treatment, and 57% self-reported needing drug or alcohol treatment. Compared with HIV-positive persons who did not inject drugs, those who injected drugs were more likely to have a detectable viral load (48% versus 35%; p = 0.008) and engage in high-risk sexual behaviors (p<0.001). Focusing on interventions that reduce high-risk injection practices and sexual behaviors and increase rates of viral suppression might decrease HIV transmission risk among HIV-positive persons who inject drugs. Successful substance use treatment could also lower risk for transmission and overdose through reduced injection.

MMP uses a two-stage sampling method. In the first stage, 23 jurisdictions are sampled from all U.S. states, the District of Columbia, and Puerto Rico. Next, simple random samples of adults with diagnosed HIV infection from sampled jurisdictions are selected from the National HIV Surveillance System, a census of persons with diagnosed HIV infection (*1*). During June 2015–May 2017, face-to-face or telephone interviews were conducted with participants, during which demographic characteristics, injection practices and sexual behaviors, and need for, and receipt of, medical services were assessed for the preceding 12 months. Response rates for 2 cycle years of data were 100% (jurisdictions) and 40%–44% (adults with diagnosed HIV infection).

Among HIV-positive persons who injected drugs, behaviors during the preceding 12 months were self-reported. Injection practices included distributive sharing of syringes and other injection equipment,* injection before or during sex, and methods for injection syringe acquisition and disposal. Participants self-reported need for, and receipt of, alcohol or drug treatment. Persons who reported receiving, or not receiving but needing, drug or alcohol treatment were considered to have a need for this service. Enrollment in a medication-assisted treatment program for opioid use disorder was also assessed. Sexual behaviors were assessed, including 1) condomless sex; 2) exchange of sex for money or goods; and 3) a dichotomous measure indicative of high risk for sexual HIV transmission (defined as having one or more detectable viral loads in the past 12 months and having high-risk sex). High-risk sex was defined as condomless sex with an HIV-negative partner or a partner whose HIV status was unknown and who was not known to be on preexposure prophylaxis (PrEP).^†^ Viral loads from the preceding 12 months were abstracted from medical records.

Weighted percentages of characteristics with corresponding 95% confidence intervals (CIs) were reported to account for complex survey design using standard methodology (*2*). Rao-Scott chi-square tests were used to compare characteristics associated with a high risk for sexual HIV transmission between HIV-positive persons who injected drugs (233) and those who did not inject drugs (7,397); p< 0.05 indicated statistical significance. All analyses were conducted using SAS (version 9.4; SAS Institute).

An estimated 3% (95% CI = 2%–3%) of persons with diagnosed HIV infection injected drugs in the preceding 12 months. Among HIV-positive persons who injected drugs, 11% engaged in distributive sharing of syringes, and 10% engaged in distributive sharing of other injection equipment; 61% injected before or during sex (Table). Common sources of injection syringes included a pharmacy or drug store (63%); a friend, relative, or sex partner (50%); a syringe services program (SSP) (32%); or a needle or drug dealer, shooting gallery, or off the street (21%). Common methods for syringe disposal were in the trash, on the street, or in a nonmedical waste container (53%); a medical waste container (50%); an SSP (30%); or keeping the syringe to reuse it (29%). An estimated 57% percent of HIV-positive persons who injected drugs reported needing alcohol or drug use treatment; 80% of HIV-positive persons who injected drugs did not obtain treatment in the preceding year. Eight percent of HIV-positive persons who injected drugs enrolled in a medication-assisted treatment program.

**TABLE:** Injection behaviors and substance use treatment in the preceding 12 months among persons with diagnosed human immunodeficiency virus (HIV) who injected drugs (n = 233) — Medical Monitoring Project, 2015–2017

Behavior/Treatment	HIV-positive persons who injected drugs	
	No.	Weighted % (95% CI)
Distributive sharing of syringes*		
Yes	22	11 (6–17)
No	204	89 (83–94)
**Distributive sharing of other nonsyringe injection equipment**		
Yes	28	10 (6–14)
No	198	90 (86–94)
**Injection before or during sex**		
Yes	141	61 (53–69)
No	87	39 (31–47)
**Reported sources of syringes^†^**		
Syringe services program	89	32 (20–44)
Pharmacy/Drug store	136	63 (54–72)
Doctor's office/Clinic/Hospital	15	5 (3–8)
Friend, relative, sex partner	111	50 (42–58)
Needle or drug dealer, shooting gallery, or off the street	50	21 (15–26)
**Disposal of syringes^†^**		
Trash/Street/Container not for medical waste	119	53 (43–63)
Kept it to reuse it	58	29 (22–35)
Put in a medical waste container	126	50 (39–61)
Took it to a syringe services program	76	30 (19–41)
**Needed drug or alcohol treatment^§^**		
Yes	134	57 (50–64)
No	99	43 (36–50)
**Obtained drug or alcohol treatment**		
Yes	40	20 (13–26)
No	193	80 (74–87)
**Enrolled in medication-assisted treatment program**		
Yes	25	8 (4–12)
No	208	92 (88–96)

* Defined as giving used injection equipment to another person for use.

^†^ Participants could report more than one response; thus, categories are not mutually exclusive, and percentages might sum to >100%.

^§^ Yes responses included all persons who self-reported a need for treatment, whether or not they received it.

A higher percentage of HIV-positive persons who injected drugs had a detectable viral load than did those who did not inject drugs (48% versus 35%; p = 0.008) (Figure). Condomless sex, exchange sex, and high-risk sex were all more prevalent among HIV-positive persons who injected drugs (63%, 17%, and 18%, respectively), than among those who did not inject drugs (31%, 2%, and 6%, respectively) (p<0.001).

**FIGURE:**
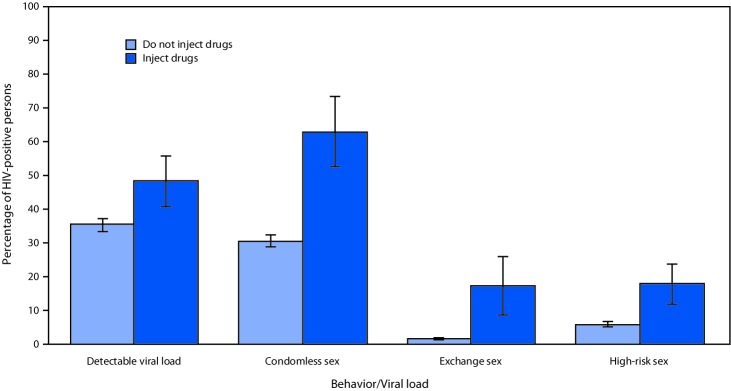
Percentage of persons with diagnosed human immunodeficiency virus (HIV) (n = 233) who engaged in high risk sexual behaviors or had a detectable viral load — Medical Monitoring Project, United States, 2015–2017 *^,^^†^ * With 95% confidence intervals indicated with error bars; all percentages are weighted. ^†^ Exchange sex was defined as exchanging sex for money or goods in the preceding 12 months; high-risk sex was defined as having one or more detectable viral load in the preceding 12 months and having condomless sex with an HIV-negative or HIV-unknown partner who was not known to be on preexposure prophylaxis.

## Discussion

Substantial high-risk injection practices and sexual behaviors associated with HIV transmission were observed among HIV-positive persons who injected drugs. Nonsterile syringe acquisition and unsafe disposal methods were common and demonstrate the need for additional outreach for harm reduction. Although a considerable need for drug and alcohol treatment was reported, 80% of HIV-positive persons who injected drugs did not obtain services, which highlights the need to expand access and referral to treatment services to reach these persons.

These findings underscore the importance of implementing a multipronged intervention approach to reducing HIV transmission among HIV-positive persons who inject drugs, including expanding access to sterile injection equipment and education regarding harm reduction and condom use (*3*). Improving access to substance use treatment might decrease HIV transmission risk through reduced need for injection. Medication-assisted treatment is one evidence-based option for opioid use disorder treatment (*4*). Viral suppression is essential for reducing HIV transmission risk and improving long-term outcomes among persons with diagnosed HIV infection (*5*). Continuing to improve retention in care and adherence to antiretroviral therapy among HIV-positive persons who inject drugs could increase the prevalence of viral suppression in this population.

Colocating HIV prevention services, such as provision of PrEP, condoms, sterile injection equipment, and HIV medical care, might also reduce the burden for patients by addressing complex public health issues in a single setting (*3*). SSPs are an important HIV prevention strategy among persons who inject drugs and could be a setting for provision of these services (*6*). Recent guidance from the U.S. Department of Health and Human Services specifies allowance of federal funds to support SSPs when there is a documented need and when the SSPs are in compliance with local laws.^§^ However, many states require legislative action to permit implementation and operation of SSPs.^¶^

A large proportion of HIV-positive persons who injected drugs received syringes from sources that provided sterile equipment, such as an SSP or a pharmacy or drug store; however, potentially nonsterile sources were also commonly used. Receipt and use of nonsterile syringes can increase the risk for acquisition of hepatitis C virus (HCV) infection among persons with HIV infection; co-infection with HCV can result in poorer clinical outcomes (*7*).**

In addition, a large proportion of HIV-positive persons who injected drugs disposed of syringes unsafely, increasing the risk of needle-stick injuries and transmission of HIV and HCV to others (*8*). Instead of disposing of syringes after first use, nearly 30% kept syringes to reuse them, which increases the risk for serious bacterial infections, including endocarditis and skin abscesses (*9*). Improving access to sterile injection equipment and harm reduction education might help to decrease the occurrence of these infections, as well as the transmission of HIV and viral hepatitis through injection equipment sharing (*6*).

The findings in this report are subject to at least three limitations. First, all characteristics ascertained through interview are based on self-report and might be subject to information bias. Second, not all sampled persons participated in MMP, but results were adjusted for nonresponse using standard methodology. Even with suboptimal response rates, results obtained using unbiased sampling methodology have value.^††^ Finally, the sample size of HIV-positive persons who injected drugs was limited; as additional data are collected in future MMP cycles, reliability of estimates should improve.

Focusing HIV prevention strategies on both high-risk sexual behaviors and injection practices among HIV-positive persons who inject drugs might reduce HIV transmission risk. Through collaborations with state and local health departments, CDC supports projects to prioritize HIV prevention strategies for persons who inject drugs. One such project, Community PROMISE,^§§^ uses peer advocates to reach persons who inject drugs and communicate public health messages around risk reduction. CDC has also expanded efforts to work with state and local health departments to detect clusters of HIV infection among important populations, including persons who inject drugs, and provides support in local investigations of these clusters.^¶¶^ CDC recommends that all persons with diagnosed HIV infection receive partner services, which includes interviews regarding sexual behaviors and injection practices, education about harm reduction interventions, and identification of sexual and injection equipment-sharing contacts, so that HIV and sexually transmitted disease testing can be offered (*10*).*** Ensuring safe methods for acquisition and disposal of syringes could decrease risks of acquiring bloodborne pathogens. CDC supports the use of SSPs as part of a comprehensive HIV prevention strategy and provides guidance on support of SSP activities (*10*). Continued efforts to reduce sexual and injection HIV transmission risk through support for expanding access to sterile injection equipment, drug treatment services, PrEP, and education around harm reduction and condom use might strengthen HIV prevention programs and directly support the national initiative to end the HIV epidemic.^†††^

**Summary**

**What is already known about this topic?**

Certain injection and sexual behaviors among human immunodeficiency virus (HIV)–positive persons who inject drugs (PWID) can increase HIV transmission risk. Successful substance use treatment could lower risk of infection and overdose through reduced injection.

**What is added by this report?**

Approximately 10% of HIV-positive PWID engaged in distributive injection equipment sharing; nonsterile syringe acquisition and unsafe disposal methods were common. HIV-positive PWID were also more likely to have engaged in high-risk sexual behaviors. Eighty percent did not receive treatment for substance use.

**What are the implications for public health practice?**

Increasing access to sterile injection equipment, drug treatment services, and education around harm reduction and condom use might reduce HIV transmission among sexual and injection partners of HIV-positive PWID.

## Footnotes

* Includes frontloading/backloading into a syringe, a process in which one syringe is used to prepare a drug solution that is then divided into one or more syringes for injection.

† https://www.cdc.gov/hiv/risk/prep/index.html.

§
https://www.hiv.gov/sites/default/files/hhs-ssp-guidance.pdf .

¶ https://www.nastad.org/maps/syringe-service-program-policy-environments-across-united-states.

** https://www.cdc.gov/hepatitis/hcv/cfaq.htm.

†† https://www.sciencedirect.com/science/article/pii/S2352146515002987.

§§ https://effectiveinterventions.cdc.gov/Files/promiseoverview.pdf.

¶¶ https://www.cdc.gov/hiv/funding/announcements/ps18-1802/index.html.

*** https://www.cdc.gov/mmwr/preview/mmwrhtml/rr57e1030a1.htm.

††† https://www.hiv.gov/federal-response/ending-the-hiv-epidemic/overview.
